# Characterization and monitoring of deltamethrin-resistance in *Anopheles culicifacies* in the presence of a long-lasting insecticide-treated net intervention

**DOI:** 10.1186/s12936-018-2557-1

**Published:** 2018-11-08

**Authors:** Madhavinadha Prasad Kona, Raghavendra Kamaraju, Martin James Donnelly, Rajendra Mohan Bhatt, Nutan Nanda, Mehul Kumar Chourasia, Dipak Kumar Swain, Shrity Suman, Sreehari Uragayala, Immo Kleinschmidt, Veena Pandey

**Affiliations:** 1 ICMR-National Institute of Malaria Research, Sector-8, Dwarka, New Delhi, 110077 India; 2 Liverpool School of Tropical Medicine, Liverpool, UK; 3 Department of Infectious Disease Epidemiology, London School of Hygiene and Tropical Medicine, London, UK; 4 Department of Biotechnology, Kumaun University, Nainital, India

**Keywords:** *Anopheles culicifacies*, Deltamethrin, Long-lasting insecticidal nets (LLINs), Piperonyl butoxide (PBO), Triphenyl phosphate (TPP), Monooxygenase, Esterase, Knockdown resistance (*kdr*)

## Abstract

**Background:**

Deltamethrin-impregnated, long-lasting insecticidal nets (LLINs) were distributed in the study area from November 2014 to January 2015 to evaluate their impact on malaria transmission in the presence of insecticide-resistant vectors. Studies were carried out in 16 selected clusters in Keshkal sub-district, Chhattisgarh State, India to monitor and characterize deltamethrin resistance in *Anopheles culicifacies* sensu lato.

**Results:**

Deltamethrin susceptibility of *An. culicifacies* decreased in a post-LLIN survey compared to a pre-LLIN survey and was not significant (p > 0.05) while, the knockdown values showed significant increase (p < 0.05). Pre-exposure to piperonyl butoxide, triphenyl phosphate showed synergism against deltamethrin (p < 0.001). Biochemical assays showed significantly (p < 0.05) elevated monooxygenases in 3 of 5 clusters in post-LLIN survey-I that increased to 10 of 11 clusters in post-LLIN survey-II, while esterases were found significantly elevated in all clusters and both enzymes were involved in conferring pyrethroid resistance, not discounting the involvement of *kdr* (L1014L/S) gene that was heterozygous and at low frequency (4–5%).

**Conclusion:**

This field study, in a tribal district of India, after distribution of deltamethrin-impregnated LLINs showed decrease in deltamethrin susceptibility in *An. culicifacies*, a major vector of malaria in this study area and in India. Results indicated development of resistance as imminent with the increase in insecticide selection pressure. There is an urgent need to develop new vector control tools, with insecticide classes having novel mechanisms of resistance, to avoid or delay the onset of resistance. Regular insecticide resistance monitoring and mechanistic studies should be the priority for the malaria control programmes to suggest strategies for insecticide resistance management. The global commitment to eliminate malaria by 2030 needs various efforts that include development of combination vector control products and interventions and few are becoming available.

**Electronic supplementary material:**

The online version of this article (10.1186/s12936-018-2557-1) contains supplementary material, which is available to authorized users.

## Background

In 2016, an estimated 216 million malaria cases were reported worldwide [1] with India contributing ≈ 1.1 million cases and 384 deaths [2]. The absence of an effective anti-malarial vaccine, spread of anti-malarial drug resistance in parasites and development of multi-insecticide resistance in mosquito vectors are key reasons for inadequate control of malaria [3].

During the years 2012–2017, a World Health Organization (WHO)-coordinated, Bill and Melinda Gates Foundation-sponsored project was conducted in 80 village clusters of community health centre (CHC) Keshkal, a tribal sub-district of district Kondagaon, Chhattisgarh State. The primary aim of the study was to find out the impact of insecticide resistance in malaria vectors on malaria burden, with a secondary aim to quantify how insecticide resistance patterns change in response to insecticide-based interventions. Chhattisgarh is a malaria-endemic state with just 2% of India’s population, but which contributed 14% of annual malaria cases in 2016 and 17% in 2017 [2]. In CHC Keshkal, deltamethrin-impregnated, long-lasting insecticide-treated nets [(LLINs) Vestergaard PermaNet 2.0] were distributed from November 2014 to January 2015. Following the distribution, 98.4% of households had at least one LLIN; 80% of households were in possession of two or more LLINs, but only 38.7% of the households met the WHO universal coverage criterion of one LLIN per two persons. LLIN usage in children under 5 years old was 81.2% and in 5–14 years age range, it was 69.8%. LLIN use by adults was lower than that of children, probably due to an inadequate number of LLINs per household [4].

*Anopheles culicifacies* (Diptera: Culicidae) is reportedly the major malaria vector in Chhattisgarh State with *Anopheles fluviatilis* of more localized importance in hilly, forested regions [5]. In Chhattisgarh State, the National Malaria Control Programme has implemented DDT and pyrethroid-based indoor residual spraying (IRS) as the primary anti-malaria vector intervention [3]. Across India continuous use of DDT in IRS for the last 5 decades led to widespread resistance in *An. culicifacies.* Malathion (organophosphate) and pyrethroids were introduced into the IRS programme in 1970s and 1990s, respectively, in this State [5] and LLINs in 2009/10. Using the recent standard WHO-criteria [6], *An. culicifacies* in most districts of Chhattisgarh State were triple resistant to DDT, malathion and deltamethrin [3]. In the study area, the deltamethrin mortality in *An. culicifacies* across the 80 clusters was 97.01% in 2014–2015, and following the LLIN distribution the mortality decreased to 83.83% in 2015–2016 (KR, pers. comm.).

For proper management of insecticide resistance and better control of malaria through vector control interventions, early detection and accurate information on status of insecticide resistance and underlying resistance mechanisms in vectors are important. The present study was conducted in 16 clusters among the abovementioned 80 study clusters. The study coincided with LLIN distribution from November 2014 to January 2015. Deltamethrin susceptibility data were generated in 16 clusters by WHO tube test during the pre-LLIN distribution period in 2014 (pre-LLIN survey) and two surveys were conducted: post-LLIN survey-I in March/April 2015 and post-LLIN survey-II in October/November 2015. Synergistic bioassays were conducted with monooxygenases, carboxylesterases and esterases specific inhibitors piperonyl butoxide (PBO), triphenyl phosphate (TPP) and S,S,S-tributylphosphorotritioate (DEF), respectively, to explore the involvement of these detoxification enzyme families in phenotype resistance to insecticide deltamethrin (0.05%), alpha-cypermethrin (0.01%) and malathion (5%). Molecular studies were performed to evaluate the association between mutations in the voltage-gated sodium channel (*kdr*, L1014F/S) and deltamethrin resistance phenotype. Previous molecular studies on *An. culicifacies* showed very low frequencies of *kdr* mutations [7, 8]. Biochemical-based enzyme assays were performed to detect the target insensitive acetylcholinesterase (*i*AChE) and detoxification enzymes esterases, and monooxygenases activities in individual mosquitoes. Combined cytological and insecticide susceptibility studies were conducted in two seasons for detecting the prevalence of sibling species composition in *An. culicifacies,* a complex of 5 species which differ in seasonal prevalence, distribution patterns, host feeding preference, and vectorial potential [9].

The overall aim of the study was to assess the impact of LLINs on deltamethrin susceptibility and to inform insecticide resistance management planning.

## Methods

### Study area and survey periods

**Fig. 1:**
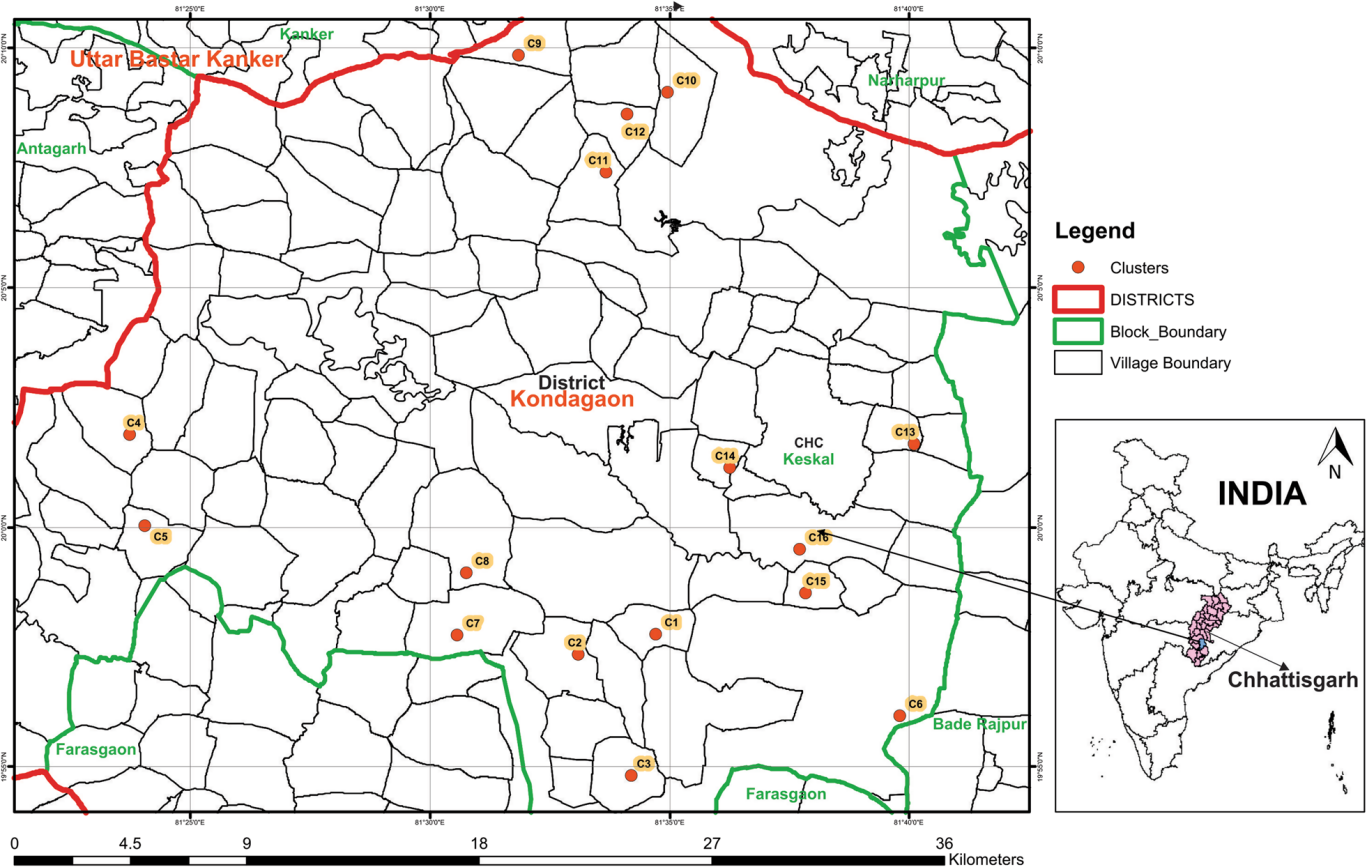
Map showing location of 16 clusters in the study area, Community Health Centres (CHC) Keshkal, district Kondagaon, Chhattisgarh State, India

The study was conducted in 16 clusters of CHC Keshkal (20°5′1N and 81°35′12E) sub-district of Kondagaon district, Chhattisgarh State, India. The 16 clusters were selected among the 80 clusters included in the project, 4 from each primary health centre based on the mosquito productivity. Malaria transmission occurs primarily during the rainy season (June to October). Vector control for the past 20 years has been a twice yearly IRS application of alphacypermethrin @ 25 mg/m^2^. The major agricultural crop in the study area is rice and pesticides used in this area include organophosphates (OP), pyrethroids and carbamates. The population in the selected 16 clusters ranged from 150 to 1100 and their geographical locations are shown in Fig. 1. Polyester LLINs (PermaNet 2.0) impregnated with deltamethrin (55 mg/m^2^) manufactured by M/s. Vestergaard Frandsen (Switzerland) were distributed in the study area from November 2014 to January 2015 in collaboration with the State health department [4]. In the present study 3 surveys were conducted in selected 16 clusters. One survey before LLIN distribution (pre-LLIN survey) in March, 2014 and two surveys after LLIN distribution in March/April, 2015 (post-LLIN survey-I) and October/November, 2015 (post-LLIN survey-II).

### Adult susceptibility tests

Adult susceptibility tests were performed following WHO protocols [6]. The blood-fed female *An. culicifacies* mosquitoes were collected by aspirator during the early hours of the day and brought to the field laboratory in cloth cages (≈ 30 cu cm) covered with wet towels. Mosquitoes were identified based on morphological characters using a standard key [10]. The WHO diagnostic dose insecticide deltamethrin 0.05%-impregnated papers were obtained from the Vector Control Research Unit (VCRU), Universiti Sains Malaysia, Malaysia (http://www.usm.my). The fully fed mosquitoes were exposed to insecticide-impregnated papers for 1 h in 3–5 replicates (15–25 mosquitoes/replicate) along with appropriate controls. Number knocked down was noted at 3- or 5-min time intervals up to 1 h exposure. After exposure mosquitoes were transferred to holding tubes with glucose-soaked cotton pad for a 24-h holding period. The holding tubes were kept in a thermocol box with wet filter paper at the bottom to maintain relative humidity (70–80%) and temperature (27 ± 2 °C). After 24 h of holding, per cent mortality was scored. The corrected per cent mortality was calculated by applying Abbott’s formula [11], if the mortality in control replicates was between 5 and 20% and if the control mortality is more than 20% the test was discarded. The susceptibility or resistance in mosquito populations was defined based on WHO criteria: 98–100% mortality indicates susceptibility, 90–97% mortality requires further confirmation of possible resistance, and below 90% mortality indicates resistance [6]. Statistical analysis was performed to determine knock-down time for 50 (KdT_50_) using log-time probit regression analysis (PASW 16.0 version) and R statistical software version 3.4.1 for further analysis of adult susceptibility data and knockdown times. Data were fitted using generalized linear mixed effects statistical models (GLMMs) to describe the effects of collection round on deltamethrin mortality and median knockdown time. For mortality data, a binomial distribution model was used. The outcomes were assessed as a function of round as a fixed effect, and collection village as a random factor. Models were chosen based upon Akaike Information Criterion.

### Synergist bioassay

Synergist bioassays were performed to assess possible involvement of insecticide resistance mechanisms in field-collected, blood-fed, female *An. culicifacies* from study clusters. Three synergists were used in this study, namely PBO (Sigma, USA) an inhibitor of monooxygenases [12], TPP (Sigma, USA) an inhibitor of carboxylesterases [13, 14] and DEF (Sigma, USA) an inhibitor of esterases [12]. Three insecticides were used deltamethrin (0.05%), alpha-cypermethrin (0.01%) and malathion (5%). The synergist (10%)-impregnated papers (12 cm × 15 cm Whatman No. 1 filter papers) were prepared in laboratory and insecticide-impregnated papers were procured from VCRU. Two treatments were compared for each test: insecticide alone and synergist + insecticide combination. In the insecticide alone, test mosquitoes were exposed to insecticide-impregnated papers for 1 h and during the synergist + insecticide combination assay mosquitoes were exposed for first 1 h to synergist-impregnated papers followed by 1 h to the insecticide-impregnated papers. After exposures mosquitoes were transferred to holding tubes for 24-h holding period and analysed as above to determine per cent mortality.

### Isolation of mosquito DNA

After the adult deltamethrin susceptibility test, live and dead mosquitoes were separated and were preserved in isopropanol for DNA extraction. Mosquito DNA was isolated by DNAzol method (Invitrogen) essentially following manufacturer’s instructions.

### L1014F/S kdr genotyping

The point mutation [Leucine (L) to Phenylalanine (F)] in codon 1014 of the voltage-gated sodium channel (VGSC) was identified by Amplification Refractory Mutation System (ARMS) following the method described by Singh et al. [15] with minor modifications. Another point mutation Leucine (L) to Serine (S) in the same codon was identified by Primer Induced Restriction Analysis PCR (PIRA-PCR) following method developed by Singh et al. [7] with minor modifications. Products were visualized on 2% agarose gel and stained with ethidium bromide (0.5 µg/ml).

### DNA sequencing

In order to validate PCR-based *kdr* genotyping, DNA sequence was performed. A part of IIS4-IIS5 linked to IIS-6 segment of VGSC was amplified by two separate PCRs following published protocol by Singh et al. [15]. Purified amplicons were sequenced by Central Instrumentation Facility (CIF), South Campus, University of Delhi, New Delhi.

### Biochemical assays

Blood-fed female *An. culicifacies* mosquitoes were collected from the study area in post-LLIN surveys-I and -II and transported to National Institute of Malaria Research (NIMR) field unit to obtain F_1_ progeny. Two- to 3-day old, sugar-fed, female mosquitoes were transported in liquid nitrogen to NIMR, New Delhi. Acetylcholinesterase, esterase and monooxygenase enzyme assays were performed following WHO guidelines [16]. Assay reaction absorbance was measured by NanoQuant Infinite^®^ M200 PRO ELISA reader (Tecan Group Ltd, Switzerland) with inbuilt Magellan 7.2 software. The results were analysed by Mann–Whitney U test.

### Native-PAGE

Native-PAGE was performed for determining α- and β-esterase profile of the susceptible laboratory and field strains of *An. culicifacies* following the procedure described by Prasad et al. [14]. The imageJ (Wayne Rasband, ImageJ 1.50i, National Institutes of Health, USA, http://imagej.nih.gov/ij/) software was used to carry out densitometry analysis of the stained native gels for estimating esterase activity.

### Sibling species identification

Field collected *An. culicifacies* sibling species were identified based on cytological method. The ovaries were removed from individual semi-gravid female mosquitoes and were stored in Carnoy’s fixative (1:3 acetic acid: methanol). Then overies were processed for preparing polytene chromosomes described by Green and Hunt [17]. The complex of *An. culicifacies* sibling species A, B, C, and D was identified based on paracentric inversions on the X-chromosome and chromosome arm 2 [9].

To study the susceptibility of sibling species to insecticides deltamethrin (0.05%) and malathion (5%), semi-gravid females were exposed to insecticide-impregnated papers for 1 h [18]. After 1-h exposure, ovaries were extracted from dead (malathion)/knockdown (deltamethrin) and live mosquitoes separately and processed as above. The per cent mortality of given sibling species was calculated based on formula given below. Susceptibility or resistance in sibling species were determined based on WHO criteria [6].

$${\text{\% }}\,{\text{mortality}}\,{\text{in}}\,{\text{given}}\,{\text{sibling}}\,{\text{species}}\, = \,\frac{{{\text{No}}\,{\text{dead}}\,{\text{of}}\,{\text{given}}\,{\text{species}}}}{{{\text{Total}}\,{\text{alive}}\,{\text{ + }}\,{\text{dead}}\,{\text{of}}\,{\text{given}}\,{\text{species}}}}\, \times \,100$$

## Results

### Adult susceptibility test

**Fig. 2:**
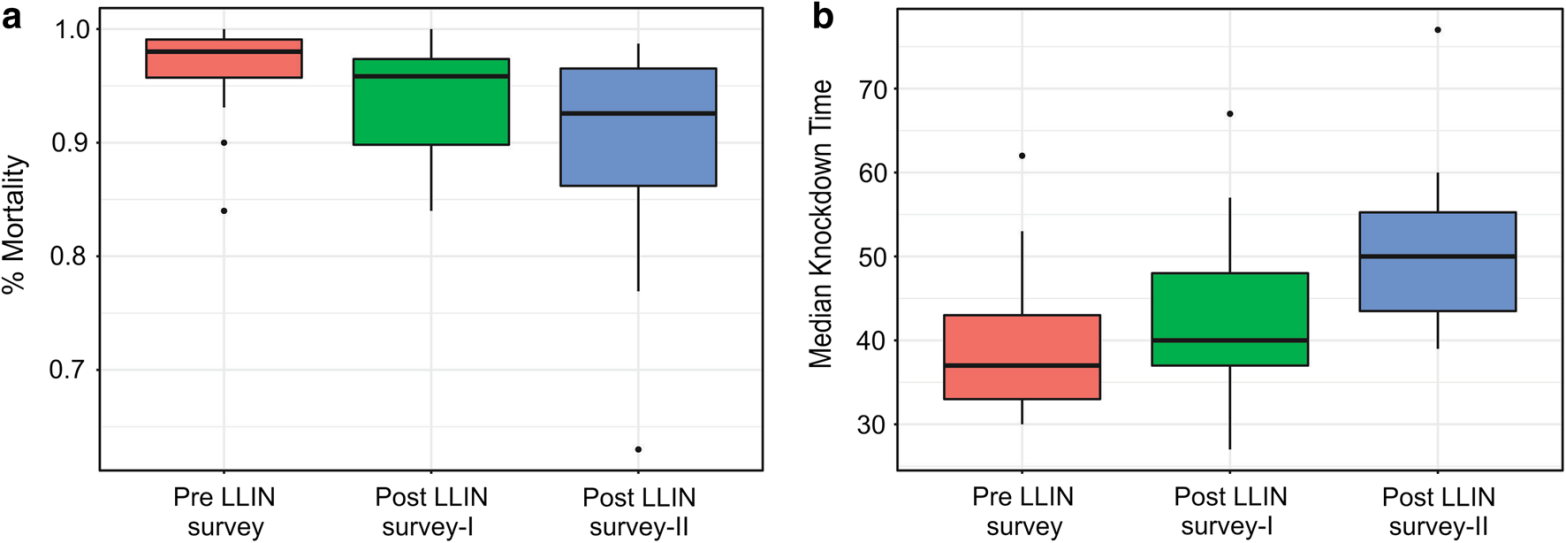
Box plots showing (**a**) mortality in *Anopheles culicifacies* (**b**) knockdown time (minutes) in the clusters in pre-LLIN survey and post-LLIN surveys-I and -II

The cluster-wise data of per cent mortality and knockdown (KdT_50_) values are given in Table 1. The cluster-specific mortalities for *An. culicifacies* to deltamethrin ranged from 62 to 100%. There was a small but non-significant (p > 0.05) decrease in mortality over the 3 surveys, pre- and post-LLIN surveys-I and -II (Fig. 2a). The knockdown indices (KdT_50_) calculated from the data in 1-h exposure showed a trend in increase in KdT_50_ values. The median knockdown time increased significantly (p < 0.05) over the 3 surveys (Fig. 2b).

**Table 1:** Susceptibility status of *Anopheles culicifacies* to deltamethrin (0.05%) collected from 16 clusters of three surveys, once in before LLIN distribution (Pre LLIN survey) and twice after LLIN distribution (Post LLIN-survey I and II)

Clusters	Pre LLIN survey (March, 2014)		Post LLIN survey-I (March/April, 2015)		Post LLIN survey-II (October/November, 2015)	
	% Mortality (n)	KdT_50_ (min)	% Mortality (n)	KdT_50_ (min)	% Mortality (n)	KdT_50_ (min)
C1	100 (91)	30	96 (113)	57	97 (103)	50
C2	95 (103)	40	97 (112)	54	98 (102)	56
C3	100 (33)	36	96 (77)	43	92 (84)	45
C4	98 (100)	33	97 (79)	27	87 (95)	59
C5	99 (105)	31	99 (115)	41	92 (81)	41
C6	99 (98)	43	97 (63)	39	91 (81)	50
C7	98 (83)	38	84 (100)	67	97 (78)	42
C8	84 (100)	62	89 (104)	57	93 (82)	48
C9	97 (67)	43	90 (70)	37	77 (91)	53
C10	99 (103)	33	97 (71)	35	82 (60)	55
C11	93 (29)	53	92 (53)	46	83 (104)	60
C12	100 (94)	31	93 (44)	37	99 (78)	39
C13	96 (98)	44	100 (49)	31	92 (94)	54
C14	99 (120)	36	89 (56)	38	62 (101)	77
C15	98 (101)	43	86 (48)	38	96 (82)	44
C16	90 (100)	36	100 (59)	42	96 (72)	40

*C* cluster, *n* number of samples, *min* minutes, *KdT*_*50*_ 50% knockdown in mosquitoes population

### Synergistic assay

**Fig. 3:**
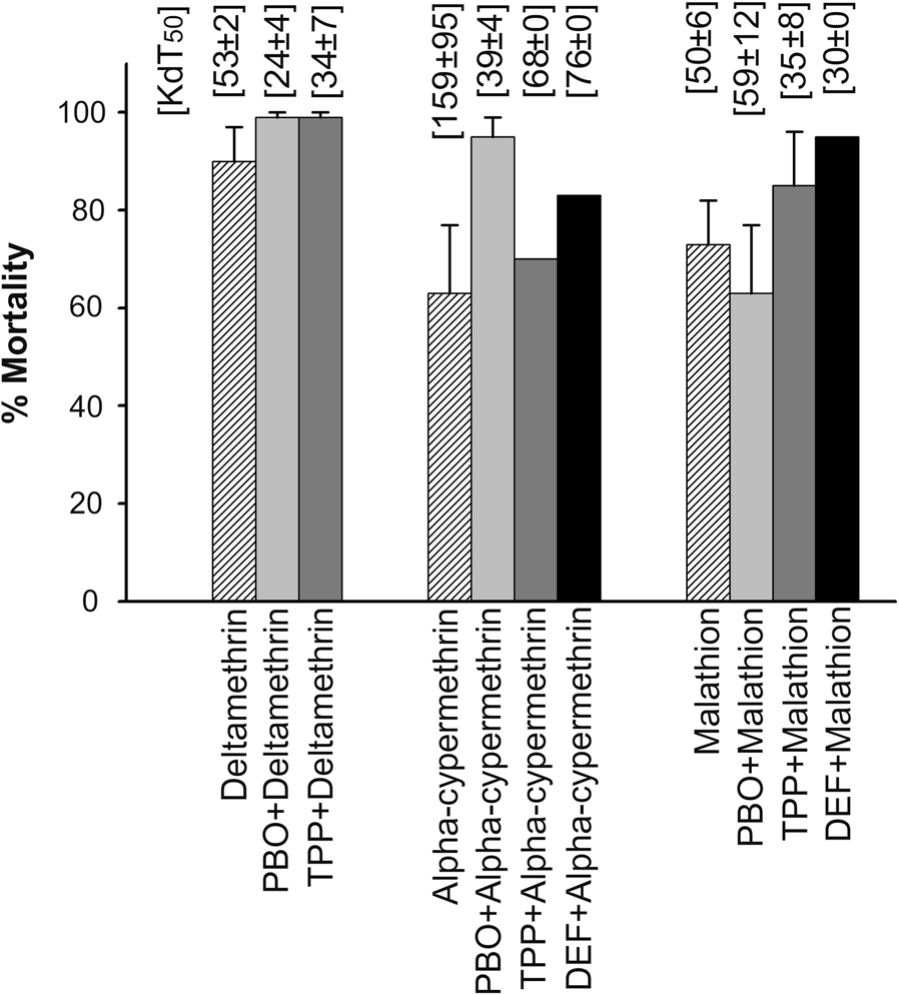
Synergistic bioassay data of pre-exposure to PBO, TPP followed by deltamethrin exposure, and pre-exposure to PBO, TPP, DEF followed by alpha-cypermethrin, and malathion exposure. Calculated KdT_50_ values in minutes

Synergistic data of exposure to synergist PBO, TPP and DEF are depicted in Fig. 3. Field-collected *An. culicifacies* mosquitoes were pre-exposed to the monooxygenases-specific synergist, PBO showed synergism to insecticides deltamethrin and alpha-cypermethrin. The average deltamethrin per cent mortalities significantly increased from 90 ± 7 to 99 ± 1 in PBO + deltamethrin exposed population compared to deltamethrin alone (p < 0.001, χ^2^ test). The knockdown times (KdT_50_) in these assays decreased from 53 ± 2 min (mean ± SD) to 24 ± 4 min at 95% CI. Against alpha-cypermethrin, the per cent mortalities significantly increased from 63 ± 14 to 95 ± 4 (p < 0.001, χ^2^ test) and was higher than those recorded for deltamethrin exposure, and KdT_50_ values decreased from 159 ± 95 to 39 ± 4 min. The PBO showed antagonism to malathion and the mean per cent mortalities slightly decreased from 73 ± 9 to 63 ± 14 (p > 0.05, χ^2^ test), and KdT_50_ values increased from 50 ± 6 to 59 ± 12 min.

Carboxylesterase-specific inhibitor TPP showed synergism to insecticides deltamethrin, alpha-cypermethrin and malathion. TPP showed similar kind of synergistic effect as PBO on deltamethrin and the average per cent mortalities increased significantly from 90 ± 7 to 99 ± 1 (p < 0.001, χ^2^ test) and KdT_50_ values decreased from 53 ± 2 min to 34 ± 7 min. For alpha-cypermethrin the per cent mortalities slightly increased from 63 ± 14 to 70 ± 0 (p > 0.05, χ^2^ test) and KdT_50_ values decreased from 159 ± 95 to 68 ± 0 min. The TPP synergism in pyrethroids may be due to esterase bonds in the structure. The average malathion per cent mortalities increased significantly from 73 ± 9 to 85 ± 11 in TPP + malathion exposed population compared to malathion alone exposed population (p < 0.001, χ^2^ test) and KdT_50_ values decreased from 50 ± 6 to 35 ± 8 min.

The non-specific esterases inhibitor DEF showed strong synergism against alpha-cypermethrin and malathion in *An. culicifacies* compared to synergist TPP. The per cent mortalities increased significantly from 63 ± 4 to 83 ± 0 for alpha-cypermethrin and 73 ± 9 to 95 ± 0 for malathion (p < 0.05, χ^2^ test) and KdT_50_ values decreased from 159 ± 95 to 76 ± 0 and 50 ± 6 to 30 ± 0, respectively.

### Knockdown resistance gene (*kdr*) frequency

The live and dead *An. culicifacies* mosquitoes from deltamethrin susceptibility tests were genotyped for two *kdr* mutations Leu-Phe (L1014F) and Leu-Ser (L1014S). Genotype association studies showed L1014F and L1014S *kdr* mutations conferred significant protection against deltamethrin in both the surveys. In post-LLIN survey-I, 408 mosquitoes (25 alive and 383 dead) were examined and there was a significant difference in codon 1014 genotypes between categories (Fisher’s exact test p < 0.0002). The 1014F and 1014S allele frequencies were, respectively, 0.12 and 0.10 in alive mosquitoes and 0.03 for both the alleles in dead mosquitoes. In post-LLIN survey-II, 490 mosquitoes (72 alive and 418 dead) were examined and again there was a significant difference in codon 1014 genotypes between categories (Fisher’s exact test p < 1.15 × 10^−6^) of the 1014F and 1014S alleles were 0.12 and 0.10 in live mosquitoes and 0.05 and 0.02 in dead mosquitoes, respectively (Table 2). The *kdr* mutations in the genome were also confirmed by DNA sequencing in 9 sequences of DNA from individual field-collected mosquitoes.

**Table 2:** Distribution of L1014, 1014F and 1014S knockdown resistance (*kdr*) alleles frequency in *An. culicifacies* collected from 16 clusters

Survey	n	Phenotype	Genotypes						Allele frequency			p value (Fisher exact test)		
			L/L	L/F	F/F	L/S	S/S	F/S	L	F	S	L vs F	L vs S	Post LLIN survey-I vs Post LLIN survey-II
Post LLIN survey-I	408	Alive	15	6	0	3	1	0	0.78	0.12	0.10	0.002	0.053	0.015
		Dead	336	21	0	26	0	0	0.94	0.03	0.03			
Post LLIN survey-II	490	Alive	48	10	3	7	3	1	0.78	0.12	0.10	0.008	0.001	
		Dead	362	38	0	17	0	1	0.93	0.05	0.02			

*n* number of samples, *p* probability value

### Acetylcholinesterase assay

**Fig. 4:**
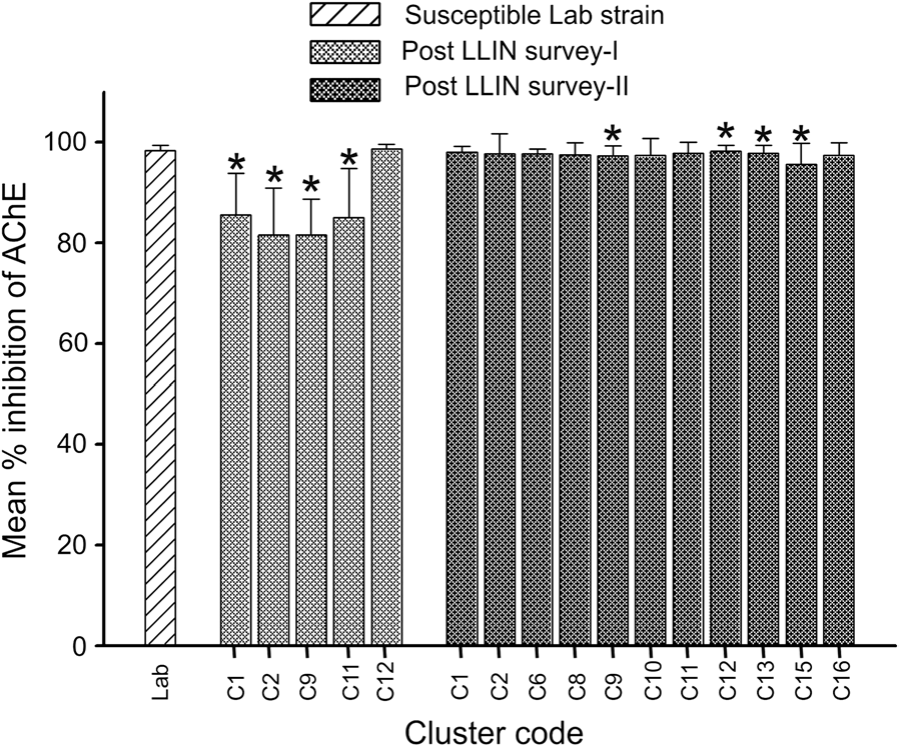
Inhibition of AChE activity by propoxur in *Anopheles culicifacies* collected from study area and susceptible laboratory strain. Asterisk % inhibition is significantly low compared to susceptible strain

The mean % inhibition of AChE was 98 ± 1.1 (% inhibition ± Standard Deviation) in susceptible *An. culicifacies* laboratory population. The AChE inhibition studies were conducted in field-collected *An. culicifacies* from 5 clusters in post-LLIN survey-I and 11 clusters in post-LLIN survey-II. The mean % AChE inhibition values during the post-LLIN survey-I were between 82 ± 7.1 and 99 ± 1 and during the post-LLIN survey-II values were between 96 ± 4.2 and 98 ± 1.2, indicating decrease in % AChE inhibition in field *An. culicifacies* population at very low frequency (Fig. 4).

### Detoxification enzymes esterases and monooxygenases

**Fig. 5:**
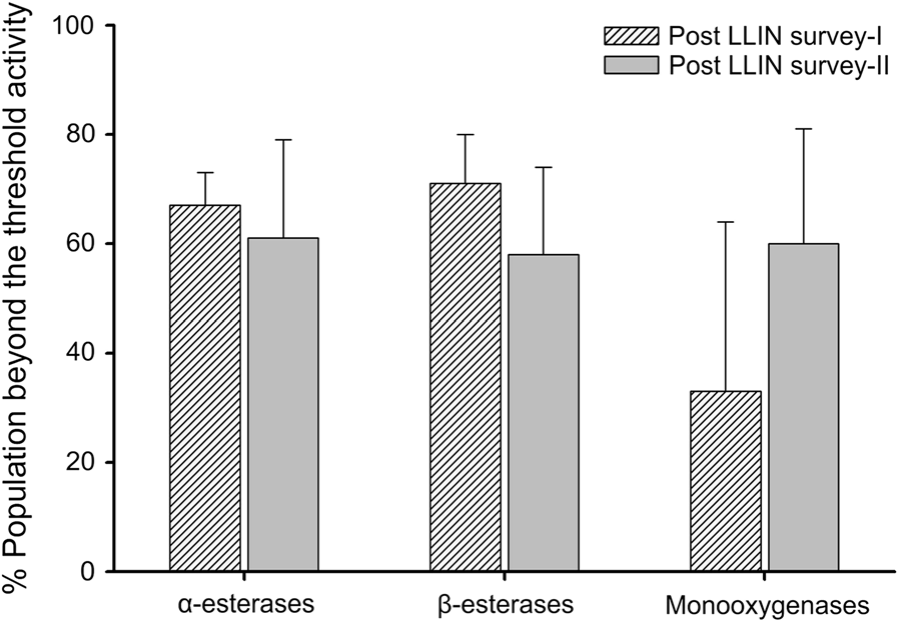
Graph showing α- and β-esterases and monooxygenases activity in *Anopheles culicifacies* population beyond the respective susceptible threshold activity

The detoxification enzymes esterases and monooxygenases, activities were determined on *An. culicifacies* samples from 5 to 11 clusters in post-LLIN surveys-I and –II, respectively. The mean α- and β-esterases and monooxygenases enzyme activities in susceptible laboratory and field populations are shown in Table 3. The results of α- and β-esterases showed significantly (p < 0.05, Mann–Whitney U test,) higher activity in *An. culicifacies* field populations in both the surveys except in one cluster (C11) for β-esterase in post-LLIN survey-II. The maxim α- and β-esterase activity in susceptible lab strain was considered as the threshold activity and was 0.92 and 0.74 mmol/min/mg. The population beyond threshold α- and β-esterase activity value were 67 and 71% in post-LLIN survey-I and 61 and 58% in post-LLIN survey-II (Fig. 5). For monooxygenases, significantly increased activity (p < 0.05, Mann–Whitney U test) levels were found in mosquito population in 3 and 9 clusters in post-LLIN surveys -I and –II, respectively. The maximum monooxygenase activity in susceptible lab strain was considered as the threshold activity and was 0.0039 mmol/mg. In post-LLIN survey-I, 33% of the population showed monooxygenase activities beyond threshold, while it was 60% in post-LLIN survey-II (Fig. 5). The results indicated elevated levels of α- and β-esterases and monooxygenases in the field population compared to susceptible counterpart.

**Table 3:** Mean α- and β-esterase and monooxygenases activities in field collected *An. culicifacies*

Survey	Site	n	α-Esterases, mmol/min/mg ± SD	β-Esterases, mmol/min/mg ± SD	Monooxygenases, mmol/mg ± SD
	Susceptible lab strain	47	0.62 ± 0.08	0.50 ± 0.09	0.0026 ± 0.0005
Post LLIN survey-I	C1	60	1.14 ± 0.56*	1.04 ± 0.53*	0.0030 ± 0.0011
	C2	60	1.27 ± 0.55*	1.27 ± 0.61*	0.0034 ± 0.0014*
	C9	53	1.50 ± 0.72*	1.38 ± 0.70*	0.0027 ± 0.0011
	C11	123	1.37 ± 0.65*	1.38 ± 0.70*	0.0031 ± 0.0013*
	C12	30	1.39 ± 0.64*	1.14 ± 0.67*	0.0064 ± 0.0024*
Post LLIN survey-II	C1	47	1.78 ± 0.79*	1.39 ± 0.68*	0.0047 ± 0.0017*
	C2	47	0.91 ± 0.47*	0.74 ± 0.43*	0.0042 ± 0.0020*
	C6	47	1.05 ± 0.57*	0.86 ± 0.52*	0.0075 ± 0.0045*
	C8	34	1.57 ± 0.77*	1.12 ± 0.70*	0.0052 ± 0.0015*
	C9	47	0.91 ± 0.54*	0.81 ± 0.52*	0.0040 ± 0.0020*
	C10	47	1.35 ± 0.72*	0.98 ± 0.62*	0.0058 ± 0.0024
	C11	47	0.90 ± 0.60*	0.72 ± 0.57	0.0051 ± 0.0016*
	C12	47	1.20 ± 0.58*	0.87 ± 0.54*	0.0040 ± 0.0022*
	C13	47	0.96 ± 0.55*	0.82 ± 0.56*	0.0045 ± 0.0016*
	C15	47	1.05 ± 0.60*	0.91 ± 0.52*	0.0034 ± 0.0013*
	C16	47	2.25 ± 1.00*	2.21 ± 1.08*	0.0065 ± 0.0018*

*n* number of samples, *SD* standard deviation

* Levels of enzyme activity significantly increased compared with susceptible lab strain

### Native-PAGE

**Fig. 6:**
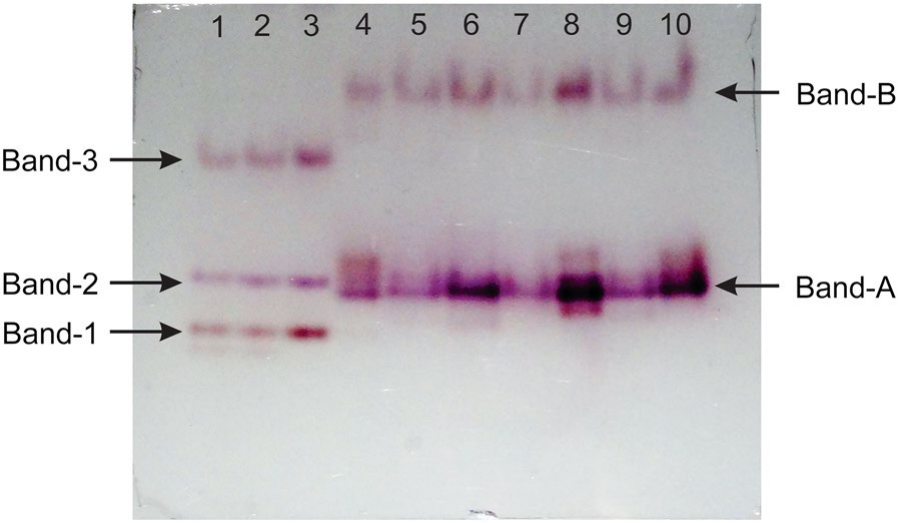
Activity of esterases on native-PAGE in *Anopheles culicifacies*. Susceptible laboratory (lane 1–3), field collected (lane 4–10)

The electrophoretic migration pattern of esterase activity in female *An. culicifacies* of susceptible laboratory strain and field population from selected clusters aged between 1 and 4-day post-eclosion are shown in Fig. 6. In susceptible laboratory strain, 3 bands (designated as Band-1, Band-2, Band-3), while in field populations, 2 bands (designated as Band-A and Band-B) were localized on PAGE by staining with ɑ- and β-naphthol acetate. The calculated R_f_ (retention factor) values for Band-1, -2 and -3 were 0.63, 0.54 and 0.30, respectively, while Band-A and -B were 0.56 and 0.17. The esterase bands were characterized as alpha/or beta esterases by the appearance of brown or purple bands on the native-PAGE. Band-1, -2 and -3 and Band-A and -B, all hydrolyze generally both alpha- and beta- naphthyl acetates though Band-2 and Band-A seems more specific to beta-naphthyl acetate hydrolysis as seen in purple colour on native-PAGE. On analysis with Image J software, the Band-A intensity of field-collected *An. culicifacies* was 2.5–10 times more than that of Band-2 intensity of susceptible laboratory *An. culicifacies*, as shown in Fig. 6, indicating possible overexpression of Band-A in field population.

### Sibling species prevalence

Distribution of *An. culicifacies* sibling species was examined among the 16 clusters, during post-LLIN surveys-I and -II. Species B and C were present in this area and % distribution of the sibling species is given in Additional file 1: Table S1. The overall sibling species B was dominant over species C (90.2 vs 9.8% in post-LLIN survey-I; 89.6 vs 10.4% in post-LLIN survey-II).

In post-LLIN survey-I the 1-h percent mortality to malathion in species B was 77% and in post-LLIN survey-II it was 66%, while in species C 1-h percent mortality was 25 and 71%, respectively (Additional file 1: Table S2). The response to malathion between two sibling species was significant during the post-LLIN survey-I (p < 0.05, χ^2^ test) while in post-LLIN survey-II, it was insignificant. Overall, it can be stated that both species B and C developed resistance to malathion.

The deltamethrin 24-h percent mortalities ranged from 62 to 100% in 16 clusters in both the surveys (Table 1). In post-LLIN survey-I the 1-h % knockdown of species B was 71 and in post-LLIN survey-II it was 100, while species C registered 67 and 100% knockdown, respectively (Additional file 1: Table S2). Thus, both species showed similar susceptibility status.

## Discussion

Development of deltamethrin resistance and resistance mechanisms were studied in the major malaria vector *An. culicifacies* from 16 clusters in tribal sub-district Keshkal of Kondagaon district in Chhattisgarh State, central India. Cohort-based active case surveillance (ACS) studies conducted in these LLIN-distributed 80 clusters by Chourasia et al. [19] reported 84% reduction in malaria incidence and sub-clinical malaria significantly in children under 14 years old. Continued use of LLINs was ensured through regular monitoring by village level, women, health volunteers and the usage of LLINs in children under 5 years old was 81.2% and children between 5 and 14 years old was 69.8% [4].

In the 16 study clusters selected for the study, adult susceptibility tests against WHO diagnostic dosage of deltamethrin (0.05%) showed decrease in susceptibility in both the surveys after LLIN distribution compared to before LLIN distribution, and was not significant (p > 0.05), while, the knockdown time values (KdT_50_) showed significant increase (p < 0.05). A 10-year study (1998–2007) in Western Uganda to assess the impact of conventionally treated insecticide-treated nets (ITNs), deltamethrin (25 mg/sq m), cyfluthrin (50 mg/sq m), and alpha-cypermethrin (50 mg/sq m) on development of resistance in *Anopheles gambiae* showed 4-fold increase in KdT_50_ values with about 1.5-fold decrease in susceptibility [20]. However, a 3-year study by Vulule et al. [21] in Western Kenya on the impact of permethrin-treated nets and curtains stated 2.4-fold increase in tolerance in the first year that did not sustain in subsequent years although reduction in parous rate and malaria transmission was observed. Such observations on variations in deltamethrin phenotypic resistance among study villages was observed owing to selection from IRS and ITNs [22]. There are conflicting observations on sustainability of pyrethroid resistance in time and space owing to selection by vector control interventions.

In this study, *An. culicifacies* showed 20–76% mortality to another pyrethroid insecticide, 0.01% alpha-cypermethrin, during post-LLIN survey-I which could be due to alpha-cypermethrin IRS in these areas in past 20 years. *An. culicifacies* registered 63–84% mortality to malathion (5%). The species has shown resistance to malathion and variable susceptibility to alpha-cypermethrin and deltamethrin. The development of deltamethrin resistance in *An. culicifacies* was earlier reported from different tribal districts of Chhattisgarh State, during studies in 2009 and 2010 by Bhatt et al. [5] with mortality in the range of 42–99% while to malathion it was 10–73%. In studies conducted during the same period (2009–2010) in 32 tribal districts of other four states: Andhra Pradesh, Jharkhand, Odisha, and West Bengal, *An. culicifacies* registered deltamethrin resistance in 4 districts, to malathion in 14 districts, while in some districts this species reported susceptibility to both the insecticides [23]; 9 tribal districts of Madhya Pradesh showed resistance to deltamethrin in 2 districts and to malathion in 7 districts [24]. In another study in 2014 in 5 tribal districts of southern Odisha, this species showed resistance to both insecticides [25]. *An. culicifacies* in the study area in district Kondagaon and other districts of Chhattisgarh State and congruent states, Odisha and Madhya Pradesh showed variable susceptibility status to deltamethrin and malathion. The pyrethroid resistance in these areas was likely due to selection by pyrethroid IRS and LLINs and possibly agriculture.

Cytogenetic studies in the study area in 2 surveys (post-LLIN surveys-I and -II) indicated prevalence of species B (90%) and C (10%). Sibling species B and C were characterized as resistant to deltamethrin in post-LLIN survey-I but susceptible in post-LLIN survey-II. In the present study the % knockdown in *An. culicifacies* increased by 12% in post-LLIN survey-II (from 71% in post-LLIN survey-I to 83% in post-LLIN survey-II) (Additional file 1: Table S2) at the end of 1 h exposure, stating an increase in susceptibility which is also reflected in sibling species. However, the proportionate increase in the sibling species could not be seen as the sample size was low for cytotaxonomical studies as only readable polytene chromosome plates could be examined that resulted in loss of samples. The sample size for species C was very low. Species B showed trend for susceptibility. Both the species were resistant to malathion in the surveys and without differences between the species. In a study conducted in Andhra Pradesh in 1980s in cash crop cultivated areas, *An. culicifacies* developed malathion resistance in the absence of malathion IRS. Species C reportedly developed resistance (4–6% mortality) to malathion faster than species B (48–76% mortality) [18]. Agricultural use of insecticides has been suggested as one of the major drivers of insecticide resistance in malaria vectors *An. gambiae* [26, 27] and *An. culicifacies* [28].

In the present study, preliminary information on metabolic resistance mechanisms was obtained using synergist bioassays; PBO showed synergism against pyrethroid insecticides, deltamethrin and alpha-cypermethrin with mortalities increased by 9% against deltamethrin and 32% against alpha-cypermethrin, and KdT_50_ values decreased 2 times and 4 times, respectively. Previous studies showed PBO synergistic effect against deltamethrin resistance in *Anopheles stephensi* from India [29], *An. gambiae* from Cameroon, Central Africa [12], *Anopheles arabiensis* from rural southeastern Tanzania [30], and *Anopheles hyrcanus* from Thailand [31].

In the present study, PBO showed antagonistic effect against organophosphate insecticide malathion and mortalities decreased by 10% and increased KdT_50_ values by 9 min in mosquitoes exposed to PBO+ malathion compared to malathion alone, exposures indicating non-involvement of monooxygenase in conferring malathion resistance in *An. culicifacies* in the study area. The current observation is consistent with previous PBO synergist studies conducted with malathion-resistant *An. culicifacies* population from Surat [13] and in *An. stephensi* from Pakistan [32].

Synergistic bioassays with TPP showed synergism against pyrethroids and organophosphate insecticides. The mortalities increased by 9% for deltamethrin, 7% for alpha-cypermethrin and by 12% for malathion, and KdT_50_ values decreased by 1.5, 2.3 and 1.5 times, respectively, in *An. culicifacies* pre-exposed to TPP. Synergist DEF for non-specific esterases showed synergism against alpha-cypermethrin and malathion insecticides and the mortalities increased by 20% against alpha-cypermethrin and by 22% against malathion, and the KdT_50_ values decreased by 2.1 and 1.6 times, respectively. DEF showed stronger synergistic effect than TPP for malathion. A study by Raghavendra et al. [13] showed carboxylesterase-mediated malathion resistance mechanism in *An. culicifacies* from Surat by TPP. In another study, Matowo et al. [30] showed moderate synergism of TPP against pyrethroid in *An. arabiensis* from rural southeastern Tanzania. In another study, significant increase in deltamethrin activity was reported in *An. hyrcanus*-resistant population from Thailand by pre-exposure to 4% PBO and 0.25% DEF [32]. In a study by Akiner and Eksi [33], PBO and DEF synergistic studies with *Culex pipiens L* showed decrease in toxicity of malathion and pyrethroids, permethrin and deltamethrin from 4 different locations in Turkey. Esterases can mediate resistance to organophosphates, carbamates and pyrethroids which are rich with ester-bonds [34]. Similarly, in the present study, pyrethroid and organophosphate resistant-*An. culicifacies* showed involvement of carboxylesterase and other non-specific esterases in conferring resistance as probable minor mechanisms.

Identification of biochemical-based resistance mechanisms using microplate enzyme assays in a single mosquito is more informative and could be of value in early detection of insecticide resistance in field population [13, 35–37]. Target site insensitive AChE assay and detoxification enzymes, ɑ- and β-esterases and monooxygenases assays were conducted in F1-female *An. culicifacies* in a few selected clusters in post-LLIN surveys-I and -II. The biochemical enzyme assay results of field samples were compared with susceptible laboratory strain of *An. culicifacies*. The AChE assay results indicated low level of AChE activity in the population. AChE is a target of 2 major classes of insecticides: OP and carbamates. In the study area, *An. culicifacies* is susceptible to carbamate insecticide bendiocarb (93 to 100%). The activities of ɑ- and β-esterases and monooxygenases significantly increased in post-LLIN surveys compared to susceptible mosquitoes. In post-LLIN survey-I, 67 and 71% of population showed ɑ- and β-esterases activity beyond the susceptible threshold value while it decreased to 61 and 58%, respectively, in post-LLIN survey-II. For monooxygenases activity, 33% of the population in post-LLIN survey-I showed activity beyond susceptible threshold value while it increased to 60% in post-LLIN survey-II. The role of esterases and cytochrome P450s in pyrethroid resistance was reported in *An. stephensi* from Dubai and India [38, 39]. Hemingway [32] reported quantitative increase of esterases in malathion-resistant *An. stephensi* from Pakistan. Safi et al. [40] reported metabolic-based mechanisms, including esterases, P450s and glutathione S-transferase (GSTs) combined with insensitive AChE in *An. stephensi* from Kunar and Nangarhar provinces of Afghanistan, and further stated that the high level of resistance was found in the Nangarhar population compared to the Kunar population due to selection of different pesticides in agriculture, and, more importantly, higher number of deltamethrin-treated LLINs were distributed in the Nangarhar population. Esterases can provide resistance to organophosphates, carbamates and pyrethroids which are rich with ester-bonds [34]. Thus, it can be stated that cytochrome P450s can mediate resistance to all classes of insecticides, increased enzyme activity can be brought about by gene amplification, upregulation, coding sequence mutations, or by a combination of these mechanisms.

Genotyping results demonstrated a significant association between *kdr* genotype and deltamethrin phenotype. Overall *kdr* frequencies were low (4-5%) but suggest that *kdr* plays a role in evolving deltamethrin resistance in *An. culicifacies* in addition to mixed-function oxidases (MFOs) and esterases. Similarly, studies by Dykes et al. [8] on *An. culicifacies* from different states in India, namely Gujarat, Chhattisgarh, Haryana and Rajasthan, *kdr* mutations were in low frequency (1.2–7.4%) and mostly in heterozygous condition, and exhibited significant protection against deltamethrin.

In the present field studies in a tribal area, the multiple insecticide-resistant *An. culicifacies* has shown a decrease in deltamethrin susceptibility owing to the use of deltamethrin-impregnated LLINs. Involvement of MFOs as major mechanism associated with esterases in conferring deltamethrin resistance in *An. culicifacies* was observed as supported by synergistic bioassays.

## Conclusion

This field study in a tribal district of India, after distribution of deltamethrin-impregnated LLINs, showed a decrease in deltamethrin susceptibility in the major vector of malaria *An. culicifacies*. Among 16 study clusters, the observed variations in mortality were not significant although the knockdown times were found to increase significantly. Monooxygenases as a major mechanism associated with esterases were found to confer deltamethrin resistance and synergized by specific synergists. The *kdr* gene frequencies was mostly in heterozygous condition and showed significant protection against deltamethrin. To suggest appropriate insecticide-reliant stratagies for insecticide resistance management in disease vectors, information on insecticide-specific biochemical resistance mechanism/s is important. This is to avoid the introduction of insecticides that have similar insecticide resistance mechanism/s that could confer cross resistance to the replaced insecticide. Results of the main study in 80 clusters suggested the continued use of LLINs in spite of developing resistance, which is imminent with the increase in insecticide selection pressure, with a caution for pro-active efforts to develop new vector control tools especially with insecticide classes with novel mechanisms of resistance [41]. To avoid or delay the onset of resistance, various strategies are propounded by Global Plan for Insecticide Resistance Management (GPIRM) [42]. The global commitment to eliminate malaria by 2030 needs immediate efforts that include establishment of infrastructure for regular insecticide resistance monitoring, development of combination vector control products and interventions for effective vector control.

## Additional file

**Additional file 1: Table S1.** Distribution of *An. culicifacies* sibling species from Keshal sub district, Chhattisgarh. **Table S2.** *An. culicifacies* sibling species response (% mortality/% knockdown) against insecticides malathion and deltamethrin at the end of 1 h exposure.

## Abbreviations

LLINs: long-lasting insecticidal nets
IRS: indoor residual spraying
ITN: insecticide-treated net
IIR: implications of insecticide resistance
PHC: Primary Health Centres
CHC: Community Health Centres
WHO: World Health Organization
SD: standard deviation
DDT: dichlorodiphenyltrichloroethane
kdr: knock down resistance
*i*AChE: insensitive acetylcholinesterase
AChE: acetylcholinesterase
PBO: piperonyl butoxide
TPP: triphenyl phosphate
DEF: S,S,S-tributylphosphorotritioate
OP: organophosphate
KdT: knock-down time
GLMMs: generalized linear mixed effects statistical models
GPIRM: global plan for insecticide resistance management
VCRU: Vector Control Research Unit
PCR: polymerase chain reaction
ARMS: amplification-refractory mutation system
PIRA: primer-introduced restriction analysis
VGSC: voltage-gated sodium channels
DNA: deoxyribonucleic acid
NIMR: National Institute of Malaria Research
ELISA: enzyme-linked immunosorbent assay
PAGE: polyacrylamide gel electrophoresis
MFOs: mixed-function oxidases
CIF: Central Instrumentation Facility
