# Supplementary material for: Characterization and monitoring of deltamethrin-resistance in Anopheles culicifacies in the presence of a long-lasting insecticide-treated net intervention
Source: Malar J. 2018 Nov 8;17:414. doi: 10.1186/s12936-018-2557-1 (PMC6225645; doi:10.1186/s12936-018-2557-1)
Supplement: Supplementary file 1 — Additional file 1: Table S1. Distribution of An. culicifacies sibling species from Keshal sub district, Chhattisgarh. Table S2. An. culicifacies sibling species response (% mortality/% knockdown) against insecticides malathion and deltamethrin at the end of 1 h exposure. [file 12936_2018_2557_MOESM1_ESM.doc]

Additional file 1: Table S1 Distribution of *An. culicifacies* sibling species from Keshal sub district, Chhattisgarh

| Cluster code | Post LLIN survey-I | | Post LLIN survey-II | |
| --- | --- | --- | --- | --- |
|  | Species 'B' (n) | Species 'C' (n) | Species 'B' (n) | Species 'C' (n) |
| C1 | 11 | 2 | ND | ND |
| C2 | 19 | 1 | ND | ND |
| C3 | 46 | 1 | 21 | 0 |
| C4 | 13 | 1 | 18 | 1 |
| C5 | 27 | 1 | 48 | 6 |
| C6 | 38 | 8 | 26 | 2 |
| C7 | 22 | 0 | 33 | 2 |
| C8 | 17 | 3 | 12 | 0 |
| C9 | 21 | 3 | ND | ND |
| C10 | 25 | 2 | 41 | 6 |
| C11 | 19 | 5 | 52 | 9 |
| C12 | 17 | 4 | 58 | 9 |
| C13 | 15 | 4 | 53 | 2 |
| C14 | 11 | 1 | ND | ND |
| C15 | 22 | 0 | 27 | 4 |
| C16 | 17 | 1 | 50 | 10 |
| Total | 340 (90.2%) | 37(9.8%) | 398 (89.6%) | 45(10.4%) |

C= cluster, ND= Not done, n= number of samples

Additional file 1: Table S2 *An. culicifacies* sibling species response (% mortality/% knockdown) against insecticides malathion and deltamethrin at the end of one hour exposure.

| Insecticide | Post LLIN Survey-I  (n) | Post LLIN Survey-II  (n) | Post LLIN survey-I | | Post LLIN survey-II | |
| --- | --- | --- | --- | --- | --- | --- |
|  |  |  | B (n) | C (n) | B (n) | C (n) |
| Malathion 5%  (% mortality) | 64.7 (159) | 67.6 (1154) | 76.66 (30) | 25.00 (4) | 65.91 (311) | 70.96 (31) |
| Deltamethrin 0.05%  (% knockdown) | 71.3 (258) | 83.5 (152) | 71.15 (52) | 66.66 (3) | 100.00 (21) | 100.00 (2) |

n= number of samples
